# Epigenetic Loss of MLH1 Expression in Normal Human Hematopoietic Stem Cell Clones is Defined by the Promoter CpG Methylation Pattern Observed by High-Throughput Methylation Specific Sequencing

**DOI:** 10.23937/2469-570x/1410031

**Published:** 2016-05-24

**Authors:** Jonathan Kenyon, Gabrielle Nickel-Meester, Yulan Qing, Gabriela Santos-Guasch, Ellen Drake, PingfuFu, Shuying Sun, Xiaodong Bai, David Wald, Eric Arts, Stanton L. Gerson

**Affiliations:** 1 Department of Pathology, Case Western Reserve University, Cleveland, OH, 44106, USA; 2 Division of Hematology and Oncology, Case Western Reserve University, Cleveland, OH, 44106, USA; 3 Case Comprehensive Cancer Center, Case Western Reserve University, Cleveland, OH, 44106, USA; 4 Division of Infectious Disease, Department of Medicine, Case School of Medicine and the Center for AIDS Research, Case Western Reserve University, Cleveland, OH, 44106, USA; 5 Department of Epidemiology & Biostatistics, Case Western Reserve University, Cleveland, OH, 44106, USA; 6 RNA Center, Case Western Reserve University, Cleveland, OH, 44106, USA; 7 Center for Stem Cell and Regenerative Medicine, Case Western Reserve University and University Hospitals of Cleveland, Cleveland, OH, 44106, USA; 8 Seidman Cancer Center, University Hospitals of Cleveland, Cleveland, OH, 44106, USA; 9 Department of Mathematics, Texas State University, San Marcos, TX, 78666, USA

**Keywords:** Mismatch repair, Epigenetics, Hematopoietic stem cells, High throughput bisulfite sequencing

## Abstract

Normal human hematopoietic stem and progenitor cells (HPC) lose expression of *MLH1*, an important mismatch repair (MMR) pathway gene, with age. Loss of MMR leads to replication dependent mutational events and microsatellite instability observed in secondary acute myelogenous leukemia and other hematologic malignancies. Epigenetic CpG methylation upstream of the *MLH1* promoter is a contributing factor to acquired loss of *MLH1* expression in tumors of the epithelia and proximal mucosa. Using single molecule high-throughput bisulfite sequencing we have characterized the CpG methylation landscape from −938 to −337 bp upstream of the *MLH1* transcriptional start site (position +0), from 30 hematopoietic colony forming cell clones (CFC) either expressing or not expressing *MLH1*. We identify a correlation between *MLH1* promoter methylation and loss of *MLH1* expression. Additionally, using the CpG site methylation frequencies obtained in this study we were able to generate a classification algorithm capable of sorting the expressing and non-expressing CFC. Thus, as has been previously described for many tumor cell types, we report for the first time a correlation between the loss of *MLH1* expression and increased *MLH1* promoter methylation in CFC derived from CD34^+^ selected hematopoietic stem and progenitor cells.

## Introduction

Complex genetic, epigenetic, and phenotypic changes in normal hematopoietic cells are required during leukemogenesis and marrow failure. While numerous individual mutations are observed, the underlying mutagenic process leading to an increased rate of mutations in hematologic malignancies and to loss of hematopoietic function is unclear. Understanding these events is critical to provide a basis for preventing underlying genomic instability and thereby reduce the risk of marrow failure and malignant transformation.

Mismatch repair (MMR) pathway dysfunction increases genomic instability as observed by increased microsatellite instability (MSI) and is an established risk factor in hereditary nonpolyposis colorectal cancer (HNPCC) [1,2]. HNPCC is commonly associated with mutations of the MMR associated mut-like-homologue 1 (*MLH1*) gene [3]. Loss of MMR and increased MSI is also a characteristic of myelodysplastic syndrome, commonly culminating in secondary leukemia and other hematologic malignancies. Additionally, a relationship between loss of *MLH1* expression, independent of mutation, and CpG methylation of the 5′ *MLH1* promoter is observed in MMR defective tumors and cell lines [4-9].

We recently determined *MLH1* expression lost occurs as a function of age in human hematopoietic progenitor cells (HPC) [10]. We observed significant MSI accumulation in the HPC and colony forming cells (CFC)s obtained from normal donors as a function of donor age. We also identified a correlation between donor age and loss of *MLH1* gene expression. We speculated acquired epigenetic changes rather than mutation was responsible for the loss of *MLH1* expression and subsequent accumulation of MSI with age.

Promoter hyper methylation of *MLH1* is associated with loss of *MLH1* expression in HNPCC [5]. The 5′ promoter region -938 bp upstream of *MLH1* transcriptional start site (position +0) is considered a CpG-rich island with 63 potential CpG sites where 5-methylcytosine (5mC) and 5-hydroxymethylcytosine (5hmC) residues are observed. While a CpG methylation of the *MLH1* promoter is known to lead to loss of *MLH1* expression and consequently functional MMR deficits, it is less clear if the specific pattern of CpG methylation has relevance to *MLH1* gene expression status. A detailed comparison of the degree and pattern of specific CpG methylated sites within the *MLH1* promoter to *MLH1* expression has not been attempted. For instance, what degree of methylation is associated with loss of gene expression? Is methylation at specific CpG sites correlated with loss of *MLH1* expression? Is the density of CpG methylation of any importance? We therefore hypothesized the frequency and pattern of CpG methylation at specific CpG residues will correlate with loss of *MLH1* expression in hematopoietic CFC clones.

To address these questions, we first identified CFC with and without detectable MLH1 expression by quantitative real time PCR (QRT-PCR). Next we determined CpG promoter methylation frequency by bisulfite sequencing multiple reads (many thousands) from single CFC by high-throughput pyrophosphate mediated sequencing. We expected sequence reads from individual CFC of normal donors would carry a spectrum of CpG methylation patterns. To identify *MLH1* promoter CpG methylation events correlating with loss of *MLH1* expression, we compared the expression status of *MLH1* in individual CFC to the frequency of methylation at each of the CpG residues in the promoter region (-938 bp to -337 bp). Our analysis defined *MLH1* expressional status of each CpG analyzed as a binary classifier input variable, i.e. expressing CFC = 1 or non-expressing CFC = 0, based on QRT-PCR results.

Unsurprisingly, classical statistical methods reveal increased methylation was associated with CFC lacking *MLH1* expression. We next analyzed the frequency of methylation each CpG residue by classification and regression tree (CART) to determine if we could predict *MLH1* gene expression status. For the first time multiplexed high-throughput bisulfite sequencing of the *MLH1* promoter has identified a correlation between the *MLH1* expression status of individual CFC and patterns of specific CpG residue in normal human HPC clones. Our data and technique now provide a baseline dataset to study progressive acquired *MLH1* loss in human adult progenitor cells.

## Experimental Methods

### Donor samples

Written informed consent regarding use of cell sample donation was obtained for all tissues used in this study under University Hospitals IRB protocol 3ZO3. Samples originate from normal heparinized bone marrow aspirates (BMA)s taken from the iliac crest or bone marrow scoop samples obtained during surgical orthopedic joint replacement procedures from otherwise healthy individuals as discarded tissue. The 30 CFC used for sequencing were selected from 4 donors out of a larger pool of donor samples on the basis of *MLH1* expression (n = 10) or lack of expression (n = 20) as measured by QRT-PCR. A list of donors and CFC used in this study is presented in table 1.

### Culture of CFC

The mononuclear cell fraction was obtained by ficolldensity gradient separation as described previously [10]. CD34^+^ cells were isolated from the mononuclear cell fraction by immune-magnetic separation with the CD34^+^ isolation kit (Miltenyi Biotech, Auburn, CA) according to the manufacturer's protocol. CD34^+^ cells were then placed in complete methylcellulose media, MethoCult H4434 Classic™ (STEMCELL Technologies Inc., Vancouver, Canada), at clonal density (33,000 cells / ml of medium) and grown for 10-14 days after which individual CFC were collected. MethoCult H4434 Classic™ contains methylcellulose, fetal bovine serum, bovine serum albumin, recombinant human stem cell factor, recombinant human GM-CSF, recombinant human IL3, and recombinant human erythropoietin and will generate CFU-E, BFU-E, CFU-GM, CFU-GEMM, and CFU-Mk colonies. The CFC subtype was not determined for colonies used in this study.

### DNA and RNA isolation

Individual CFC were washed with PBS and cells divided into two equal fractions. Genomic DNA was obtained from one fraction of the cells obtained from an individual CFC by proteinase K digestion as previously described in [10]. Briefly one fraction of the CFC was washed twice with PBS and then incubated at 55°C for 2 hours in 75 ul lysis buffer (10mM Tris-HCl (pH 8.0), 1mM EDTA, 1% Triton-X 100, and 10 mg/mL proteinase K). Following lysis proteinase K was inactivated by incubating for 10-minute at 80°C. RNA was isolated from the second fraction with the RNAqueous-Micro Kit™ (Ambion Inc., Grand Island, NY). Between 50 and 100 ng of total RNA was used to generate cDNA with the Superscript III First-Strand Synthesis kit (Invitrogen, Carlsbad, California) with random hexameric primers as described in the manufacturer's protocol.

### MLH1 gene expression by QRT-PCR

QRT-PCR analysis of human *MLH1* gene expression was performed with the TaqMan™ gene assays for human *MLH1* (assay #Hs00179866_m1; Applied Biosystems, Foster City, CA) and compared relative to human β*-Actin* (#4352339E; Applied Biosystems, Foster City, CA). QRT-PCR analysis was performed using the Standard mode of a 7500 Fast Real-Time PCR system (Applied Biosystems, Foster City, CA). We analyzed samples in triplicate. Cycle threshold value was calculated in order to determine relative expression (RQ) between samples. RQ values for *MLH1* were calibrated relative to expression observed in the K562 cell line. CFC for which amplification of β-Actin was above the cycle threshold value but lacked amplification over cycle threshold for *MLH1* were scored negative for *MLH1* expression. Threshold values were optimized automatically by the Applied Biosystems 7500 Fast Real-Time PCR system analysis software to fall within the amplification exponential phase of all samples, with the exception of MLH1 non-expressing CFC as MLH1 expression in these CFC could not be detected above baseline. Samples expressing detectable amounts of *MLH1* template were given the binary classifier score of 1 (*MLH1* expressing) while CFC samples lacking detectable *MLH1* template were given the binary classifier score of 0 (*MLH1* non-expressing). The cycle thresholdsgenerated by the Applied Biosystems 7500 Fast Real-Time PCR system analysis software for β-*Actin* and *MLH1* were 0.34 and 0.10 respectively.

### Methylation specific sequencing of the MLH1 promoter

**Figure 1:**
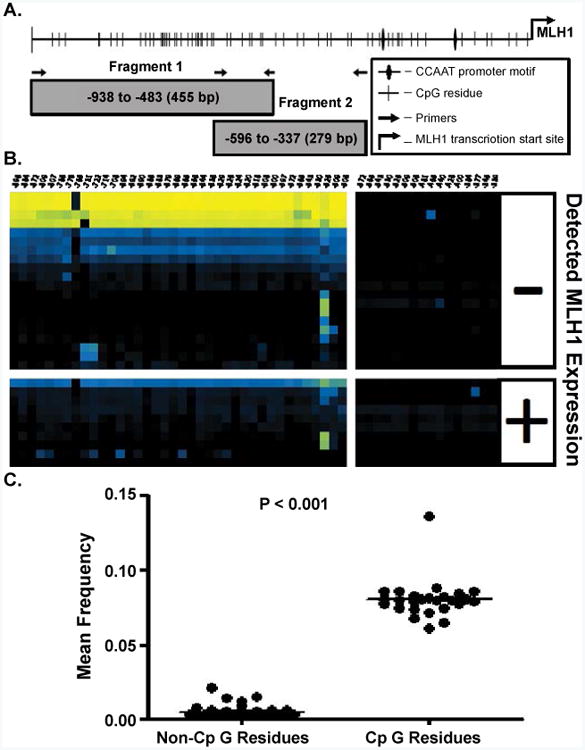
A) An illustration of the MLH1 promoter region identifying the MLH1 transcriptional start site, CpG residues, CCAAT box, and primer binding locations. CpG sites are numbered from the transcription start site located at position 0, NCBI sapiens chromosome 3 genomic contig, GRCh37.p9 Primary assembly Reference Sequence NT_022517.18 Fragment 1 CpG residues are located at: -896, -884, -872, -809, -807, -786, -776, -765, -731, -722, -714, -708, -694, -692, -690, -686, -683, -679, -669, -665, -656, -644, -636, -629, -626, -624, -620, -618, -608, -600, -597, -572, -565, -543, -530, -525, -509, and -506 bp and Fragment 2 CpG residues are located at: -572, -565, -543, -530, -525, -509, -506, -481, -465, -449, -428, -400, -384, -377, -345, and -339. B) Bias corrected CpG methylation frequency is depicted as a heat map, each block representing the frequency of methylation at a single CpG within a single CFC sample. The methylation frequency at CpG residues are read from right to left along horizontal axis. Each row represents a unique CFC and each column represents a specific CpG. The frequency scale is generated with ( 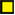) yellow is equivalent to a frequency of 1.0, blue ( 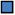) a frequency of 0.5 and black (■) a frequency of 0.0. C) A T-test comparison of the mean frequency of all non-CpG site methylation events to the total frequency of methylation at CpG residues.

Genomic DNA obtained from individual CFC was bisulfite modified with the EpiTect Bisulfite Kit™ (Qiagen, Valencia, CA) in accordance with the product protocol. Immediately following bisulfite modification, DNA from either CFC or total CD34+ cell isolate was amplified with either the MLH1-1f and MLH1-1r primer pairs to generate Fragment 1 (-938 to −483 bp) and MLH1-2f and MLH1-2r primer pair to generate Fragment 2 (-596 to −337 bp), figure 1A & table 2. Primers were designed to amplify a 601 bp region starting -938 bp upstream of the *MLH1* transcription start site (position = 0) based on the NCBI Homo sapiens chromosome 3 genomic contiguous sequence, GRCh37.p9 Primary Assembly Reference Sequence NT_022517.18.

Products of 455 bp (Fragment 1) and 255 bp (Fragment 2) obtained following the first round PCR amplification were gel extracted and amplified in a second round PCR amplification with fusion primers each containing the 454 universal adapter A and B sequences and the multiplex identifier (barcode) sequences necessary for individual sample identification and multiplexing, i.e. each barcode was a unique sequence and identified a specific CFC. Approximately equal molar pools of Fragment 1 and 2 products were next run on a 454-GS FLX+ system™ (454 Life Sciences Corporation, Branford, CT) pyrosequencer by the Farncombe Metagenomics Facility in the Health Sciences Center, McMasters University to obtain bidirectional single molecule sequence reads.

## Sequence Analysis

Sequence reads from 454 run data were first identified and sorted by barcode, aligned to the theoretical bisulfite modified Fragment 1 and Fragment 2 sequences, filtered for sequences with > 70% identity, and finally CpG site methylation was scored for methylation with a Perl script written by Dr. Bai. Sequence reads were each compared to a theoretical bisulfite converted genomic consensus sequence. Non-methylated CpG sites (observed as a CA sequence) were scored as 0 while methylated CpG sites (observed as a CG sequence) were scored as 1. Non-CpG C→T conversions were also scored to determine the theoretical bisulfite conversion efficiency. The data discussed in this publication have been deposited in NCBI's Gene Expression Omnibus and are accessible through GEO Series accession number GSE73868 http://www.ncbi.nlm.nih.gov/geo/query/acc.cgi?acc=GSE73868) [11].

## Methylation Frequency Bias Correction

Methylation frequency at each CpG site was determined for each CFC. Hyperbolic bias correction was performed as described in [12] with the use of enzymatically methylated and non-methylated control DNA at ratios of 1:0, 1:1, and 0:1. A corrective hyperbolic solution was next calculated and applied to the methylation frequency of each CpG residue. The bias corrected methylation frequencies were then normalized to a value between 0 and 1 (between 0-100% methylated), see supplemental table 1.

## Classical Statistical Analysis

Calculations for linear regressions and unpaired T-tests were performed with GraphpadPrizim 4 version 4.03© software (Graphpad Software La Jolla, CA). Logistic regression was performed with the PROC LOGISTIC of the STAT module of the SAS statistical software package (SAS Institute Inc., Cary, NC).

## Classification and Regression Tree (CART) Analysis

The methylation frequency values of CpG sites were used as predictive variables for a CART model of *MLH1* expressing or non-expressing CFC under the assumption methylation is a factor involved in loss of *MLH1* expression (Splus, TIBCO Software Inc., Palo Alto, Calif.) as described in [13,14]. Recursive partitioning based on bias corrected CpG site methylation frequencies produced progressively more homogenous CFC groups. Internal segregating nodes are referred to as branches and terminal nodes as leafs. Terminal nodes were created when further classification failed to improve segregation.

Our model attempts to classify two classes of CFC, *MLH1* expressing (Exp) or non-expressing (Non-Exp), thus:

k=Exp,Non−Exp

The deviance (D) of a whole classification tree was defined as a sum over all leaves (terminal nodes).

$$D=\sum_{i}D_{i},$$

If, for each node, all CFC within the node are of the same class e.g. *MLH1* expressing or non-expressing, then the value of deviance is 0 and considered optimal. Alternatively, deviance was considered maximal when CFCs classified within a node were 50% expressing *MLH1* and 50% not expressing *MLH1*. In this sense, the function of deviance is similar to the entropy equation:

$$\sum p_{ik}\log p_{ik}$$

The CART generation algorithm then uses the categorical measurement of CpG site methylation frequency as criteria for the generation of branch decisions. At each step, a node was split into two more homogenous subgroups (terminal leaves) in an optimal way through the minimization of deviance. Splitting variables were identified based on an exhaustive search of all possible branch points. Branch point construction continues until the number of cases reaching each leaf is small (we chose < 10) or the leaf is sufficiently homogeneous. A value of 1% deviance of the root node was chosen. After this preliminary tree was produced, redundant nodes were “pruned” to prevent an over-fit model. “Pruning” consisted of removal of sub-trees found to be unimportant. Akaike's information criterion (AIC) is an estimation of information lost when any single model is selected over a set of models. In this sense each “pruned tree” may represent a better model of the data. By determining the following cost-complexity measure:

$$D_{k}(T)=D(T)+k.\text{size}(T,)$$

Where *D_k_*(*T*) is the deviance or AIC of a subtree *T, size*(*T*) is the number of terminal nodes of *T*, and *k* is the cost-complexity parameter. Each potential model (“pruned tree”) is based in part, on different values of *k* thus, we calculated an estimate of divergence, then chose the model (“pruned tree”) with the lowest AIC value. Our estimation set a value of *k* = 2 at a minimal value of AIC.

## Results

### Generation of a high-throughput library of single molecule *MLH1* promoter sequences from *MLH1* expressing and non-expressing normal human CFC

To determine if CpG promoter methylation was associated with loss of *MLH1* expression in individual human HPC, we first identified CFC from four normal donors as having or lacking *MLH1* expression by QRT-PCR. *MLH1* expressing CFC were defined as those CFC in which *MLH1* and β-Actin product amplification was detected. While *MLH1* non-expressing CFCs were those CFC in which QRT-PCR amplification products were detected for β-Actin but not for *MLH1*. Thirty CFC were chosen in total as candidates for high-throughput bisulfite sequencing of the *MLH1* promoter, table 1.

DNA obtained from individual CFC was bisulfite modified and PCR products were generated for both Fragment 1 (-938 to -483 bp) and Fragment 2 (-596 to -337 bp) in an adaptation of the methods presented in [9]. An illustration of the CpG sites in *MLH1* promoter region is provided in figure 1A. A secondary PCR was performed to add linker sequences and a 9 bp unique identifying sequence for each CFC as described in the Experimental Methods section, supplementary table 1. Bisulfite modification alters unmethylated cytosine residues to uracil residues and results in inefficient plus and minus strand genomic DNA hybridization. Methylation residues of CpG sites are palindromic, thus, the primers used in bisulfite sequencing will only amplify the template strand in one direction. Our primers were designed to amplify and evaluate the CpG methylation of the minus strand of the *MLH1* promoter, figure 1A.

Sequences generated were aligned to a theoretical 100% methylated reference sequence and filtered to only include sequences with greater than 70% homology. Single molecule *MLH1* promoter Fragment 1 and Fragment 2 sequences were generated from both ends of the secondary PCR products. A total of 199,807 Fragment 1 and 111,039 Fragment 2 sequences were generated. Sequences generated from Fragment 2 suffered from a comparatively large amplification bias for unmethylated template and thus the conclusions drawn from Fragment 2 in this study were limited. Frequency of methylation at each CpG residue was calculated and corrected for bias as described in the Experimental Methods section and [12]. Characterization of CpG methylation status in a third fragment, more proximal to the MLH1 transcriptional start site (between -337 and 0 bp), yielded insufficient sequence reads for analysis, data not shown. The reason for this remains unclear, though amplification bias for unmethylated sequence reads is suspected. Additionally, all of the sequence reads described in this manuscript are accessible through NCBI's Gene Expression Omnibus Database, accession number GSE73868.

After bisulfite modification, unmethylated CpGs on the negative strand of genomic DNA are detected as cytosine: adenosine pairs when sequenced in the positive direction while a methylated CpG are observed as a cytosine: guanine pairs [15]. Thus, CA→CG conversions indicate negative strand methylated cytosines. Cytosines not followed by a guanine immediately in the 3′ direction i.e. CC, CA, and CT are identified as non-CpGcytosines. Non-CpGcytosines are not expected to be methylated in adult human samples [16]. Determination of A→G conversion frequency of minus strand non-CpGcytosines indirectly measures bisulfite conversion efficiency. The overall C→U conversion rate at non-CpG residues in our data was 99.99%; imparting a theoretical limit the detection of false positive methylation at any given cytosine to less than 0.01% (Figure 1C).

### Loss of MLH1 expression correlates with increased frequency in MLH1 promoter methylation in normal human CFC

**Figure 2:**
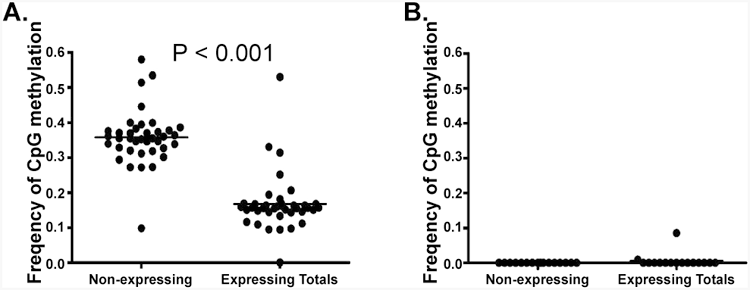
T-test comparison of the average frequency of CpG methylation at the CpG residues of MLH1 non-expressing CFC (n=20) compared to the average CpG methylation at the CpG residues of expressing CFC (n-10) in A) Fragment 1 and B) Fragment 2. Expressing CFC have a significantly lower average frequency of CpG methylation than observed in MLH1 non-expressing CFC.

Using Sanger sequencing, we previously identified a correlation between microsatellite instability and *MLH1* promoter methylation in a small number of normal HPC [10]. In this large high-throughput bisulfite sequence library of the *MLH1* promoter, sequences from each CFC were “tagged” with a unique barcode. This barcode allowed a correlative comparison between *MLH1* promoter methylation sequence and *MLH1* expression status. *MLH1* promoter methylation correlates with a loss of detectable *MLH1* expression similar to observations made in cancer cells [2, 4-7,17,18]. Within the Fragment 1 sequences, the average CpG methylation frequency at CpG sites in *MLH1* non-expressing CFC was greater than that of CpG sites in *MLH1* expressing CFC (p < 0.001) by two tailed T-test; while no statistical difference was observed within the Fragment 2 sequence set (Figure 2A,B). The average frequency of CpG methylation at individual CpG sites within Fragment 1 of non-expressing CFC was greater than that of expressing CFC in 46% of CpG residues, (p < 0.05 by two tailed T-test for individual CpG residues). Methylation status of 54% of individual CpG sites within Fragment 1 could not predict CFC *MLH1* gene expression alone. By logistic regression, the frequency of methylation at individual CpG residues was also incapable of predicting *MLH1* expression status in either Fragment 1 or 2. Methylation frequency at each CpG site of Fragment 1 sequences between *MLH1* expressing and non-expressing CFUs was examined by two-tailed T-test. Individual CpG site methylation frequency correlated with loss of *MLH1* expression at CpG sites located -896, -884, -872, -776, -731, -722, -692, -686, -683, -669, -656, -624, -618, and -608 bp upstream of the *MLH1* promoter start site (p < 0.05).

### Specific CpG site methylation frequency sort CFC by classification and regressive tree analysis

Complex CpG methylation patterns potentially play a role in gene expression and influence transcription. Establishing how genetically linked methylation sites are associated with loss of *MLH1* expression was feasible using high-throughput bisulfite pyrosequencing technology. Our methylation specific sequencing consistently resulted in read lengths longer than 400 bp for Fragment 1. This allowed a correlative comparison between observed CpG methylation patterns and the *MLH1* expression status of individual CFC. Other deep sequencing approaches (Illumina or Ion Torrent) would not have provided sufficient read lengths to perform this analysis.

To establish a correlative model, a classification and regression tree (CART) analysis was selected. CART analysis is a methodology derived from set theory and consists of the recursive partitioning of binary outcomes (*MLH1* expressing or non-expressing CFC) on the basis of potentially dependent variable information (in this case site specific CpG methylation frequency). CART analysis is often presented as a decision tree model of branch points which separate heterogeneous sets or classes into smaller, more homogenous classes [14]. The methodological basis for CART is to compare all possible pairs of variables in one class (in this case frequency of CpG site methylation for each CFC) to all other pairs of variables in another class and thus, identify an optimal methylation frequency threshold which best segregates the outcome values (i.e. the *MLH1* expression status of individual CFC). Decision branch points or internal nodes are created when a methylation frequency decision threshold inequality produces a terminal node with the lowest number of misclassified outcomes. Thus, a CART analysis, due to the recursive calculations performed, is capable of revealing potentially complex pattern specific correlations within large and complex data sets not normally accessible to traditional statistical methodology.

Tree Analysis with Randomly Generated and Evolved Trees (TARGET) is an alternative partitioning methodology. The TARGET method for generating dichotomous rules sets begins with randomly generated trees that are then recursively refined by assessments of fitness. Following each generation of refinement, the best fit trees of the previous generation are compared to newly generated random trees and to randomly and non-randomly modified versions of the best fit trees. In this way successive generations of models are tested with the expectation that over a large enough sampling of decision trees, the TARGET algorithm will generate an optimal dichotomous decision tree with minimal branch nodes and maximal fitness. CART analysis on the other hand suffers from potential bias introduced by sequentially searching for locally optimal solutions. Thus, trees built with CART may miss greater fitness trees with fewer nodes because of strong locality optimizations [19]. However, while the TARGET methodology may have yielded an algorithm with better fit, we show that a CART generated algorithm is sufficient to reasonably classify MLH1 expressing and non-expressing CFC based on CpG site methylation frequency.

**Figure 3:**
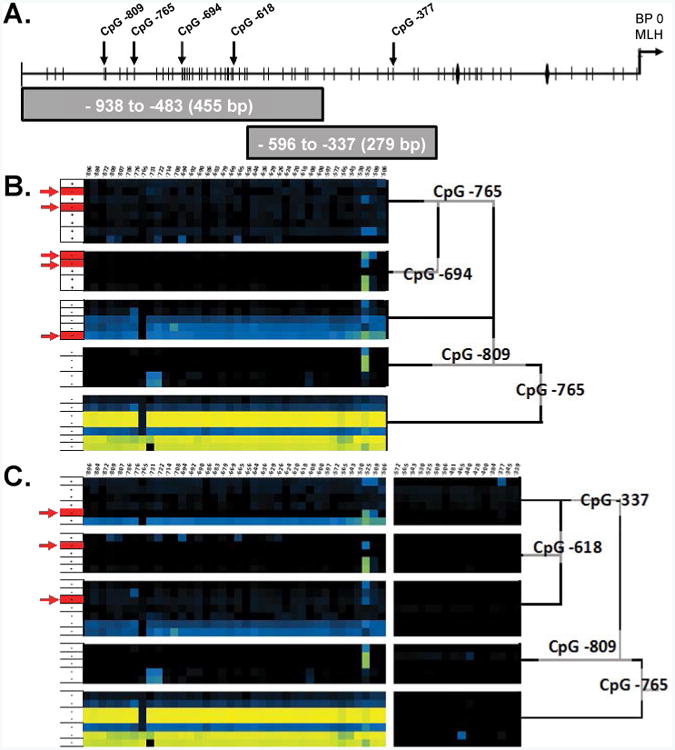
A) An illustration of the MLH1 promoter region identifying the MLH1 transcriptional start site, CpG residues, CCAAT box, and primer binding locations. CART analysis of Fragment 1 B) and the combination of both Fragment 1 & 2 C) showing clustering of similar CpG methylation frequency patterns. Red arrows(→) indicate miss identified CFC and Black vertical arrows(↓)indicate CpG residues identified by CART analysis.

**Figure 4:**
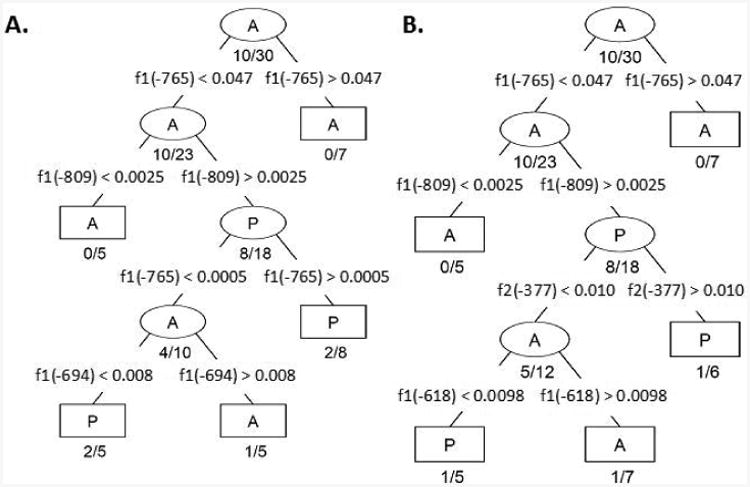
CART Decision algorithms generated with CpG methylation frequencies from **A)** Fragment 1 (f1) and **B)** Fragment 2 (f2) combined. Each branch node (ellipse) defines a branch point which filters CFC into progressively more homogenous classes. The terminal nodes (rectangles) indicate no further partitioning is necessary (either the size of the node is small or the node is sufficiently homogeneous). Branch nodes are labeled with the majority CFC expression identity; as labeled with an A to indicate the majority CFCs classified within a node lack MLH1 expression while P indicates the majority of CFC expressed MLH1. Misclassification ratio is indicated below each branch and terminal node. The segregating parameter is indicated by the CpG residue location followed by an inequality statement and CpG methylation value indicating the optimal threshold between the two nodes.

We present a model consisting of a dichotomous set of rules optimally predicting the *MLH1* expression status of CFC based on the similarity of methylation frequency observed at the CpG sites within Fragments 1 and 2 (Figure 3 A-C). To determine this dichotomous set of CpG site methylation frequency rules, we performed CART analysis of bias corrected CpG site methylation frequencies from individual CFC either expressing or not expressing *MLH1*. Then, as described in the Experimental Methods section, determined CpG methylation frequency rules which optimally differentiated the 30 CFC into classes of methylation frequency patterns that either expressed or did not express *MLH1* [13]. We initially utilized only sequences from Fragment 1. This analysis indicated the CpG methylation frequencies observed for a CFC at CpG -765, -809, and -694 bp were able to sort individual CFC into five classes (Figure 3A,B). This classification successfully predicted expression status of CFC with an 83% success rate. Inclusion of Fragment 2 CpG site methylation frequencies further improved in the CART analysis classification success. CART analysis with CpG methylation frequency data for both Fragment 1 and Fragment 2 determined the methylation frequencies of CpG at -765, -809, -377, and -619 could segregate CFC into 5 distinct classes of CFC. The combined Fragment 1 and Fragment 2 CART analysis resulted in a successful classification rate of 90%. The first two decision branches made in this analysis were at CpG -765 and -809 and were identical to the CART algorithm in which only Fragment 1 CpG methylation frequency was considered (Figure 4A,B).

## Discussion

Promoter methylation of the *MLH1* gene is observed in leukemia and lymphomas as well as in *MLH1* deficient human colon, endometrial, and gastric tumors [20-24]. Studies of *MLH1* deficient tumors have primarily focused on discovery of mutation target sites due to loss of MMR, the presence of MSI, and the clinical course of these diseases. Examples of *MLH1* CpG promoter methylation in the tissue of normal donors are limited. Additionally, our analysis identifies methylation as a correlating factor in hematopoietic precursor cells. While we accept this study does not link loss of *MLH1* expression and promoter methylation to tumorigenic mutation; it does represent the identification of a predisposing processes of *MLH1* expression loss in the CFC from otherwise normal individuals and is, therefore, a significant finding. We previously reported the appearance of MSI in normal human hematopoietic progenitor cells increases with age [10]. Additionally we found a correlation between CFC with evidence of MSI, loss of *MLH1* expression, and increased *MLH1* promoter methylation. This led us to hypothesize loss of *MLH1* expression in normal hematopoietic stem and progenitor clones might be associated with *MLH1* promoter methylation.

The field of bisulfite sequencing has advanced dramatically over the last decade. However, even with the utilization of high-throughput deep sequencing technology, this technique is limited by an inability to detect a difference between 5mC and 5hmC residues. It is possible our assessment of promoter methylation of the *MLH1* gene detected 5hmC residues. The effect 5hmC residues might have on *MLH1* expression is currently unclear. There is conflicting evidence as to whether 5hmC is a precursor to a demethylation, a unique epigenetic modification, or both. The ten eleven translocation (TET) methylcytosine dioxygenase class of enzymes catalyze the conversion of 5mC to 5hmC but are incapable of converting unmethylated cytosine residues [25]. In global genomic analyses of TET function throughout embryogenesis, TET expression corresponded to accumulation of 5hmC and a loss of 5mC. Following DNA synthesis any newly formed daughter DNA strands containing complementary CpG to a parental 5hmC residue are not modified by the DNA methyltransferase DMNT1 to a 5mC. This phenomenon results because DMNT1 is incapable of recognizing 5hmC as methylated and subsequently cannot maintain 5hmC methylation palindromic sequences on newly formed daughter DNA strands. Thus, 5hmC residues are likely lost as a result of replication-coupled dilution [26-29]. Neurons within the central nervous systems of mammals, however, largely do not replicate. Indeed 5hmC accumulation is in fact observed within neurons and likely plays an important role in the epigenetic regulation of gene expression within the brain [30]. So then, if 5hmC accumulation is limited by replication-coupled dilution one would not expect to observe 5hmC in a replicating population of HPCs in the act of forming a CFC as TET methylcytosine dioxygenase activity would be limited by the availability of 5mC residues which, upon conversion to 5hmC cannot be maintained in replicating cells.

Given the variability in promoter methylation, the numerous CpG sites at risk, and the lack of appreciation as to whether CpG methylation density or methylation of specific sites impact gene expression, we undertook the current study. We characterized CpG methylation events from −938 bp to -337 bp of the *MLH1* promoter of single hematopoietic stem or progenitor cell clones from normal human donors by clone-specific multiplexed high-throughput single molecule bisulfite sequencing and correlated this dataset to the *MLH1* expression status of each individual CFC. Similar to tumor cell line *MLH1* expression and promoter studies we found increased CpG promoter methylation correlated with a loss of *MLH1* expression. This is the first study to identify a correlative relationship within clonal expansions generated from a CD34^+^ enriched population of otherwise normal human adult hematopoietic stem and progenitor cells. Further we identify a subset of CpG sites associated with disruption of *MLH1* expression, as has been found in other settings [31,32]. These observations substantiate conclusions made in our previous study [10], and provides further evidence that *MLH1* promoter methylation in normal HPC is a factor in the loss of *MLH1* expression.

Methylation sequencing studies of the *MLH1* promoter in human tumor samples and adjacent normal tissue [2,4-7,9,17,18,22-24,31-33] show similar losses of *MLH1* expression. However, in contrast, our analysis focused on single normal hematopoietic stem and progenitor cell clones, rather than bulk tumor isolates or cell lines, as is canonically studied. *MLH1* expressing and non-expressing CFC show a significant trend towards greater CpG promoter methylation in CFC lacking detectable *MLH1* expression. The mean frequency of CpG methylation in the *MLH1* promoter region observed in non-expressing CFC was nearly 38%, much greater than identified in *MLH1* expressing CFC.

Our study also provides further evidence *MLH1* promoter methylation is involved with loss of *MLH1* expression and our previous observations, in [10], of MSI in human hematopoietic stem and progenitor cell clones potentially suggest a general underlying mechanism for acquired genomic instability. Whether and how HPCs progress to hematopoietic failure or other hematologic abnormalities cannot be inferred from this data. However, the many reports of human disease associated with *MLH1* methylation and MSI suggest a causal link does indeed exist [22,34-43]. Further, since epigenetic loss of *MLH1* is implicated as a factor in spontaneous and secondary hematopoietic malignancies [44-47], it is reasonable to posit *MLH1* promoter methylation has some role in the malignant transformation of normal HPCs. Prospective monitoring of individuals identified with high rates of *MLH1* promoter methylation and assessing the presence of *MLH1* promoter methylation in HPCs prior to or early in disease evolution would be necessary to definitively illustrate the clinical relevance of *MLH1* promoter methylation on the evolution of hematologic disorders. No such study has been completed for humans to date; however, in mice, the functional consequences MMR failure is dramatic with observations of increased incidence of lymphoid tumors, hematologic malignancies and hematopoietic failure commonly observed [48-50].

Most commonly, we observed CFC lacking *MLH1* expression had increased *MLH1* promoter methylation. However, examples of CFC without *MLH1* expression occasionally were observed with low levels of promoter methylation. While our dataset largely lends support to *MLH1* promoter methylation as being responsible for loss of *MLH1* expression in differentiating HPC, other mechanisms could explain the broad variability in methylation status observed in our *MLH1* deficient CFC. Work by Sun *et al*. [51], for example, compared the transcriptomes, global histone-modifications, and DNA methylation of a highly purified population of young and old mouse hematopoietic stem cells. This work demonstrated that reduced gene expression was often associated with suppressive histone modification, CpG island methylation, or both with increased age [51]. Our work did not assess histone modifications of the *MLH1* promoter, however, we speculate that increased CpG methylation occurring more proximal to the *MLH1* transcriptional start site, histone modification, or both could contribute to this discrepancy.

Lineage commitment of hematopoietic cells is known to be associated with widespread global changes in CpG methylation [52-56]. Analysis of *MLH1* gene expression with Stanford University's Gene Expression Commons [57] demonstrates *MLH1* expression is reduced (but not lost) in both common myeloid progenitors as well as in granulocyte monocyte progenitor cells but not in common erythroid progenitors. While CD34^+^ selection does enrich for a population of hematopoietic stem and progenitor cells, the colony forming assay (from which the CFC selected for this study were derived) induces erythroid and myeloid lineage differentiation. Additionally, the specific CFC subtype was not determined for the CFC used. It is possible that through selection bias for colonies either expressing or lacking *MLH1* expression we unintentionally chose colonies expanded from either common myeloid progenitors or granulocyte monocyte progenitors and excluded common erythroid progenitor derived CFC. Thus, it is possible the loss of *MLH1* we observe is representative of a lineage programmed reduction of MMR through promoter methylation. This would however, imply that lineage committed cells would possess uniformly methylated or unmethylated *MLH1* promoters. We actually identify significant methylation heterogeneity within individual CFCs. Our observations of mixed methylation status would seem to conflict with the idea that loss of *MLH1* is a consequence of lineage commitment. However, since we cannot rule out the possibility that the selected CFC were derived from multiple progenitor cells (presumably with different methylation patterns) and because CFC subtype was not determined for the CFC selected in this study, we cannot definitively determine if our observation of *MLH1* loss is attributable to lineage commitment CpG methylation differences. The analysis of *MLH1* promoter methylation pattern in different committed progenitor cells and different CFC subtypes would be necessary to determine if lineage commitment is critical for *MLH1* expression and while intriguing is beyond the scope of this preliminary study.

Early analysis of mutation in human hematopoietic differentiated cells by Jones et al., 1995, [58] and Akiyama et al., 1995, [59] determined the frequency of inactivating mutations at hypoxanthine phosporibosyl-transferase (HPRT) in T lymphocyte clones of normal human donors over the human life span. The frequency of mutation at HPRT was nearly 10 fold greater in clones from elderly donors. Similar observations have been made regarding reduced double strand break repair capacity in aging human CD34^+^ cells [60]. Progressive loss of genes regulating genomic stability is proposed as a consequence of aging [50,61]. Acquired MMR failure in the form of MSI has previously been detected in peripheral lymphocytes and CD34^+^ cells of adult humans [10,62]. The rate of functional MMR loss observed in peripheral blood lymphocytes of HNPCC patients was greater per year in individuals with heterozygous inactivating mutations of *MLH1* or *MSH2* than age matched normal individuals [63]. Additionally, while studying the effect of defective MMR in *MSH2* deficient mice on hematopoietic function, Reese et al. observed a competitive repopulation defect in *MSH2* deficient mice [64]. Of note, detection of MSI in the HPC of these mice could only be observed following serial transplantation. By extrapolation, similar findings in hematopoietic stem and progenitor cells would be consistent with the concept of a clonal evolution of HPC leading to hematopoietic disorder. Our study did not find nor seek to determine if donor age was associated with patterns of *MLH1* promoter methylation and loss of *MLH1* expression. However, our data is consistent with the acquisition of *MLH1* promoter methylation in otherwise normal adult HPC. Given the consequences of losing MMR on genomic stability it is reasonable to speculate that aberrant promoter methylation of *MLH1* in HPC over a lifetime, could precipitate hematopoietic dysfunction and possibly increase tumorigenic potential. However, determining if donor age was associated with the acquisition of specific CpG methylation patterns in human HPC is beyond the scope of this report; though remains an attractive topic for further study.

## Supplementary Material

1

## Figures and Tables

**Table 1:** Donor CFC number, barcode, and corresponding sequence frequency generated.

					Fragment 1			Fragment 2		
Sample ID	Clone	Donor Age	Barcode sequence	MLH1 Expression	Methylated Molecules	Unmethylated Molecules	CpG Methylation Ratio	Methylated Molecules	Unmethylated Molecules	CpG Methylation Ratio
BMA01	BMA01-C13	73	ATATCTCAA	-	315	2175	0.145	106	4706	0.023
BMA01	BMA01-C14	73	ACTAGGTCC	-	726	1922	0.378	185	4220	0.044
BMA01	BMA01-C2	73	CCACGGCCG	-	494	1173	0.421	63	1907	0.033
BMA01	BMA01-C3	73	TCCTTAGCT	-	2829	8051	0.351	198	3721	0.053
BMA01	BMA01-C4	73	GAGACGTAA	-	2945	13298	0.221	483	2891	0.167
BMA01	BMA01-C8	73	GTTATGTAT	-	1400	2578	0.543	289	3385	0.085
BMA01	BMA01-C1	73	GTTTACAGT	+	2807	8399	0.334	1118	5777	0.194
BMA01	BMA01-C11	73	CAATCCCTC	+	299	1710	0.175	65	2599	0.025
BMA01	BMA01-C5	73	ACGGCCCTA	+	1292	5065	0.255	500	9305	0.054
BMA01	BMA01-C7	73	TTGCTAGGT	+	1548	7087	0.218	1634	6205	0.263
BMA02	BMA02-T1C1	42	GAGTAGGCA	-	2810	3381	0.831	139	2570	0.054
BMA02	BMA02-T1C4	42	TACGCTGGA	-	855	978	0.874	290	1092	0.266
BMA02	BMA02-T1C6	42	GGGCCATTG	-	171	439	0.390	20	374	0.053
BMA02	BMA02-T1C8	42	CGTGTACGC	-	254	801	0.317	47	846	0.056
BMA02	BMA02-T2C7	42	GACACCGGT	-	3751	3841	0.977	60	1346	0.045
BMA02	BMA02-T2C8	42	ACCAACCTT	-	3883	3968	0.979	52	1153	0.045
BMA02	BMA02-T3C8	42	TACAGGTTT	-	805	2976	0.270	147	2172	0.068
BMA02	BMA02-T1C12	42	GTACTCATG	+	297	4544	0.065	93	2114	0.044
BMA02	BMA02-T1C9	42	ACTCCGAGT	+	225	779	0.289	45	750	0.060
BMA03	BMA03-T1C1	47	TCTCCACAG	-	928	7605	0.122	137	4139	0.033
BMA03	BMA03-T2C1	47	TGAGCATGG	-	1301	6712	0.194	175	5350	0.033
BMA03	BMA03-T2C4	47	CAGAGTGTT	-	830	6879	0.121	80	3174	0.025
BMA03	BMA03-T2C5	47	AACCGCGTT	-	1354	7333	0.185	48	2573	0.019
BMA03	BMA03-T2C9	47	TCTAATGTT	-	844	4334	0.195	172	3073	0.056
BMA03	BMA03-T1C5	47	AACCCAAGA	+	1224	5789	0.211	113	3275	0.035
BMA03	BMA03-T2C7	47	TCCTTCTGG	+	6301	13041	0.483	166	2503	0.066
BMA04	BMA04-C13	74	GTAGCCTCG	-	2233	6501	0.343	516	3064	0.168
BMA04	BMA04-C14	74	AATGGCTTA	-	11820	37208	0.318	387	2595	0.149
BMA04	BMA04-C6	74	TGCCGGATA	+	1609	5991	0.269	429	2590	0.166
BMA04	BMA04-C7	74	CCCAAGGTG	+	2063	5976	0.345	474	2787	0.170

**Table 2:** Primer sequences used. A-linker adapter forward and reverse sequences are followed by a 9 bp unique [BARCODE] and Fragment 1 or 2 specific forward or reverse primer.

Name	Sequence 5′ to 3′
MLH1-1f	ACTCAAAATCCTCTACCTTATAATATC
MLH1-1r	TTAAAAGAAGTAAGATGGAAG
MLH1-2f	ACAAACCAAACACAAAACCCCAT
MLH1-2r	TTTAGTTAATAGGAGTAGAGATG
A-linker adapter forward	CGTATCGCCTCCCTCGCGCCATCAG[BARCODE][MLH1-1f or MLH1-2f]
B-linker adapter reverse	CTATGCGCCTTGCCAGCCCGCTCAG[BARCODE][MLH1-1r or MLH1-2r]

## Abbreviations

5hmC: 5-hydroxymethylcytosine
5mC: 5-methylcytosine
AIC: Akaike's Information Criterion
BMA: Bone Marrow Aspirate
CART: Classification And Regression Tree
CFC: Hematopoietic Colony Forming Cell Clone
DNA: Deoxyribonucleic Acid
HNPCC: Hereditary Nonpolyposis Colorectal Cancer
HPC: Hematopoietic Progenitor Cell
HPRT: Hypoxanthine Phosporibosyl-Transferase
MLH1: Mut-Like-Homologue 1
MMR: Mismatch Repair
MSI: Microsatellite Instability
QRT-PCR: Quantitative Real Time Polymerase Chain Reaction
RNA: Ribonucleic Acid
RQ: Relative Expression
TET: Ten Eleven Translocation
UCB: Umbilical Cord Blood
